# The contribution of medical educational system of the College of Medicine, and Health Sciences of the University of Gondar in Ethiopia on the knowledge, attitudes, and practices of graduate students of Health Sciences in relation to the prevention and control of nosocomial infections during the academic year of 2018

**DOI:** 10.1186/s12909-020-02271-6

**Published:** 2020-10-22

**Authors:** Tigist Engda

**Affiliations:** Department of Microbiology, School of Biomedical and Laboratory Sciences, College of Medicine and Health Sciences, University of Gondar, P. O. Box: 196, Gondar, Ethiopia

**Keywords:** Nosocomial infection, Health sciences students, Knowledge, Attitude, Practice

## Abstract

**Background:**

Nosocomial infection, also called a hospital-acquired infection, is an infection acquired during admitting patients in health care facilities. Nosocomial infection can be prevented and controlled by giving training to those responsible. This study aimed to assess the contribution of the medical education system on the knowledge, attitudes, and practices of the graduate students of health sciences about the prevention and control of nosocomial infection in the College of Medicine and Health Sciences at the University of Gondar in the Academic Year of 2018.

**Method:**

An institution-based cross-sectional study was conducted among all graduate health science students posted in the different departments at the University of Gondar in the College of Medicine and Health Sciences from February to June 2018. A total of 422 study participants were included. Data were analyzed using SPSS version 20.

**Results:**

Out of a total of 422 respondents, only 40% have taken training for infection prevention; out of which 39% had taken the training for a year ago. Moreover, only 35.5% have good knowledge of nosocomial infections as a result of the training; and only 32.5% have good understanding of the practical training given on prevention and control. Only 36% have good attitude towards its prevention and control.

**Conclusion:**

The result shows that only a few of the respondents have taken the infection prevention training. Yet, a smaller proportion of them had good knowledge, attitude, and practice on nosocomial infections. Hence, the Medical Education System should give more attention to the training of the nosocomial infection control by developing different strategies to prepare the students on these issues before they start their clinical attachment.

## Background

Nosocomial infection is a localized or systemic infection that is acquired in a health care facility that may manifest 48 h after the patient’s admission to or discharged from the health care facility [1]. It can be caused by bacteria, viruses, parasites, and fungi that may be present in the air, surfaces, or equipment surrounding the health institutions [2]. It can affect patients of all age groups; however, neonates, immunocompromised adults, and the elders are the most vulnerable ones [3, 4].

Nosocomial infections have been recognized as a problem affecting the quality of health care services and are the principal source of adverse healthcare effects. Increased hospital stays, increased costs of healthcare, economic hardship to patients, and their families, and even deaths are some of the negative effects [5–7].

The amount of nosocomial infections of low- and middle-income countries is higher because of the limited knowledge and utilization of Post Exposure Prophylaxis (PEP), limited knowledge of professional risks, low adherence to universal precautions (UP), and inaccessibility of personal protective equipment (PPE) [2, 3, 5, 8]. Findings of several epidemiological studies show that HCWs, mainly physicians, dentists, laboratories, and nurses are involved in the transmission of nosocomial infections [8–11]. It has also been reported that its transmission increases during the performances of medical procedures whenever HCWs fail to follow aseptic precautions [8, 10].

The World Health Organization (WHO), in conjunction with the CDC, gives high attention to the prevention of nosocomial infections as it has developed a practical manual for the prevention of nosocomial infections globally (WHO, 2002). Some recommended strategies included in the manual were the use of hand decontamination, personal hygiene, utilization of personal protectives, and proper methods of handling soiled clothing when healthcare workers perform patient care activities. It also recommends methods of preventing environmental contamination including the cleaning of the hospital environment using hot superheated water, sterilizing patient equipment, and preventing the transmission of pathogens like HIV, hepatitis B and C viruses, and TB to the staff [1].

Efficient pre-service and in-service training given by incorporating in the medical education system supported with good monitoring and evaluation methods of HCW practices play a pivotal role in the sustainability of the knowledge, attitude, and practice of universal precautions and infection control [12, 13]. This scheme was supported by many studies conducted worldwide. For example, in India, an educational module had effectively elevated the knowledge, attitude, and practice score of HCWs from 14% before the intervention to 94% thereafter [14]. In Korea, it was also investigated that a group of nurses and medical students who had received education on HAIs showed high knowledge (*P* = 0.036) and performance (*P* < 0.01) levels [15]. Similarly, a study at Seton Hall University in New Jersey, USA, indicated that the total score for the knowledge category was 93% [16]. Likewise, a study in Egypt reported that physicians had the best level of knowledge, but the least in practicing general safety measures than others in the pre-intervention phase. However, they increased their practice score from 54.3 to 86.1% after receiving continuing education [14]. A study in Pakistan reported that the knowledge score was 3.8 with a median of 4. The dispenser had the highest knowledge score while the housekeepers had the lowest. Knowledge about the mode of transmission of bloodborne pathogen and the work experience alone significantly predicted the use of universal precaution methods in multiple linear regression models [17].

This principle is also supported by studies conducted in Ethiopia. In 2015, a cross-sectional study was conducted in Addis Ababa on HCWs who received training on transmission, vaccination, and diagnosis of HBV to assess their knowledge of risk factors for HBV. The result showed that more than 80% of the respondents had the knowledge of the modes of transmission and prevention of HBV; 83.3% had a positive attitude towards following infection control guidelines [18].

Similarly, a study conducted in Ethiopia at Dire Dawa University on the medical and health sciences students reported that almost all of the respondents had good knowledge of the transmission, treatment, and prevention of HBV. Also, 94.2% of them had good attitudes towards the importance of standard precautions, but 58.4% had poor practices in applying the recommended standard precautions [19]. In another study, a hospital-based cross-sectional investigation was conducted among 150 health workers who were taking training about infection and prevention of hospital-acquired infections at Debre Markos Hospital in Ethiopia. The results showed that 84.7% of them were found to be knowledgeable; however, only 57.3% of the respondents demonstrated good practice in infection prevention. Moreover, respondents with older age, longer work experience, and higher educational status excelled in both knowledge and practice of infection prevention. In-service training, availability of infection prevention supplies, and adherence to infection prevention guidelines were also associated with the practice of infection prevention [19]. On the other hand, a study conducted at the University of Gondar Hospital about hand hygiene compliance on 405 study participants showed that only 8.97% had the knowledge about it and 5.62% had received training about hand hygiene compliance, respectively [20].

Hand hygiene is an important means for the control and prevention of nosocomial infection. Therefore, the current study intended to determine the impact of the medical education system on the knowledge, attitude, and practice of graduate health sciences students about the prevention and control of nosocomial infections at the University of Gondar.

## Methods

### Study design, area and period

An institution-based cross-sectional study was conducted with graduate students from the College of Medicine and Health Sciences at the University of Gondar. Data collection was made between February and June of 2018.

### Population

Students who attended the regular academic program at the University of Gondar, College of Medicine and Health Sciences were the source population. However, only graduate classes of Health Science students were taken as the study population.

### Sample size determination and sampling techniques

**Fig. 1:**
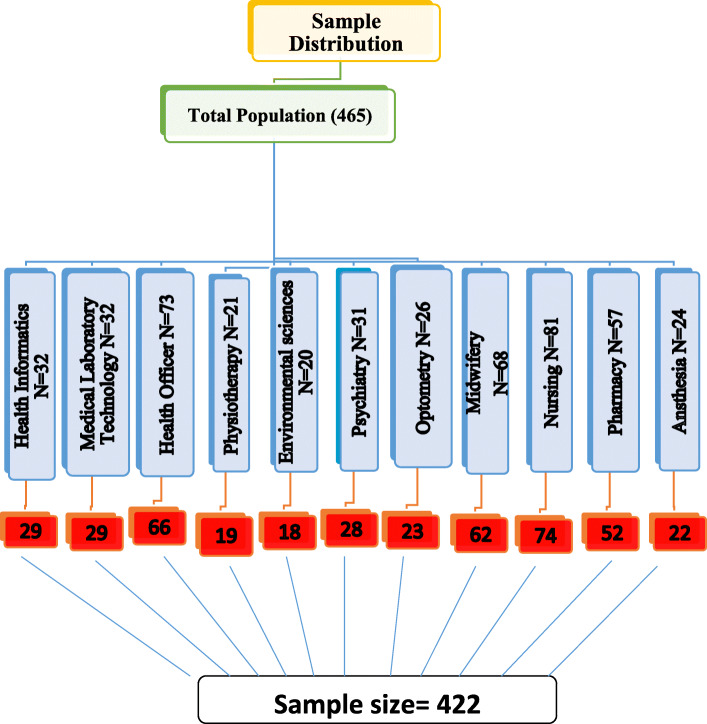
Schematic presentation of the sampling procedure

Before data collection, eleven departments were selected for sampling: Health Informatics, Medical laboratory Sciences, Health Officer, Physiotherapy, Environmental and Occupational health and safety, Psychiatry, Optometry, Midwifery, Nursing, Pharmacy, and Anesthesia. There were 465 graduate students of health sciences in the academic year of 2018. The sample size was determined using a single random sampling method. Since no similar study was found in the area, 95% were taken as a confidence interval. Then, the calculated sample size was 384 and by adding a 10% non-response rate, the final calculated sample size became 422 (Fig. 1).

The questionnaire was constructed from emergent themes reviewed in the literature and items were derived from the established guidelines set by a Task Force Committee on Infection Control Practices Advisory Committee [5, 21]. The questionnaire includes 40 questions subdivided into four categories: socio-demographic, knowledge, attitude, and practice towards nosocomial infections. Knowledge was assessed using 11 questions containing three alternative choices each. The answers from the given alternatives were symbolized as ‘1’ for poor, ‘2’ for fair, and ‘3’ for good. A higher score in the questions concerning nosocomial infections is considered to be good knowledge of nosocomial infections.

Attitude was measured using 8 questions in which answers for each question were assigned as 1 for ‘disagree’, 2 for ‘neutral’, and 3 for ‘agree.’ Higher score achieved was considered as a positive attitude toward standard precaution. Moreover, their practice towards the prevention and control of nosocomial infections was assessed using 14 questions with three alternative answers which were assigned as ‘1’ for poor, ‘2’ for fair, and ‘3’ for good (Table 1, Table 2, and Table 3).

**Table 1:**
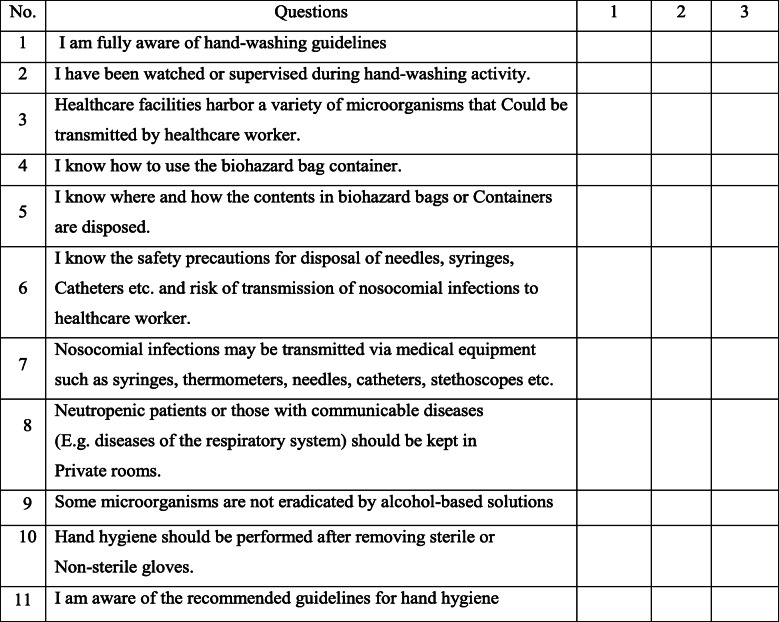
Questioner for the assessment of knowledge of graduate health sciences students towards nosocomial infection Instruction: To complete this section, please make a tick “✓” on the number corresponds to how you agree with the given alternatives 1 = Poor, 2 = Fair and 3 = Good

**Table 2:**
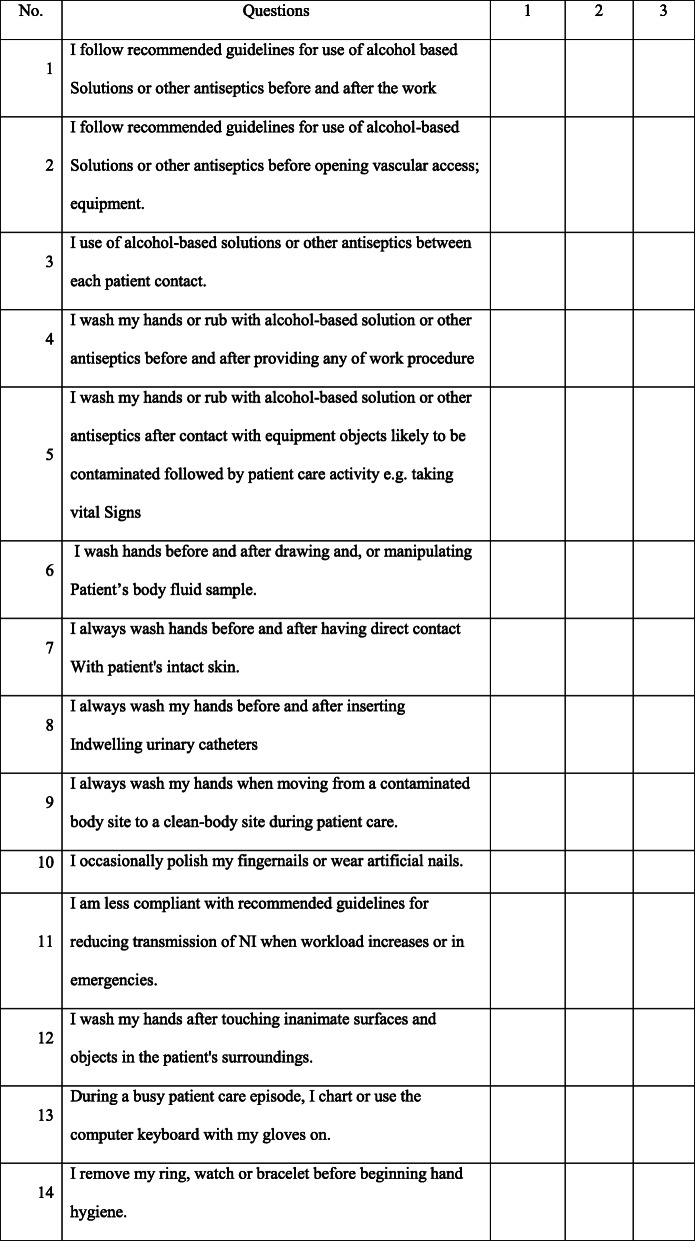
Questioner for the assessment of practice of graduate health sciences students towards nosocomial infection Instruction: To complete this section, please make a tick “✓” on the number corresponds to how you agree with the given statement 1 = Poor, 2 = Fair, 3 = Good

**Table 3:**
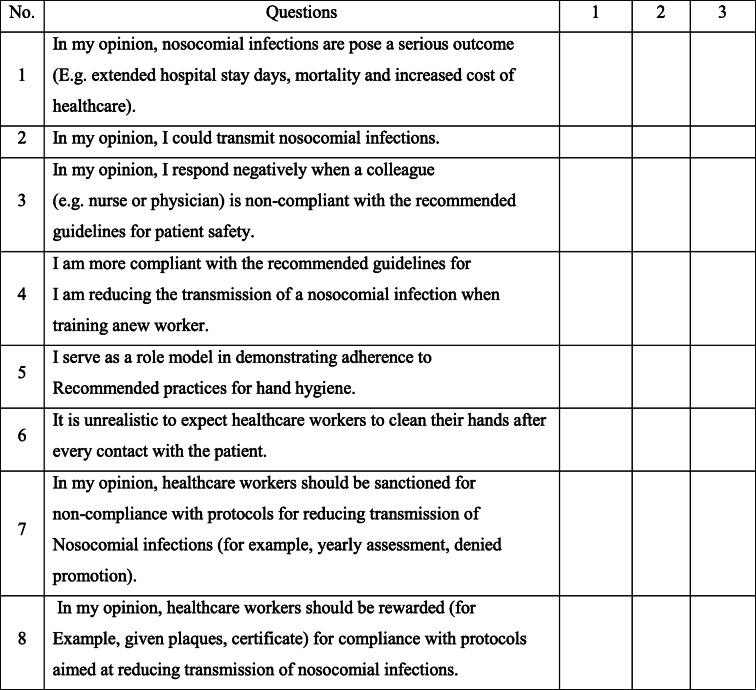
Questioner for the assessment of attitude of graduate health sciences students towards nosocomial infection Instruction: To complete this section, please make a tick “✓” on the number corresponds to how you agree with the given statement 1 = Disagree, 2 = Neutral, 3 = Agree

Bloom’s cut-off point was used to determine the level of Knowledge, attitude, and practice because the conceptual framework of the present study was based on the taxonomy of educational objectives developed by Bloom (1956).

According to Bloom’s taxonomy (1956), human behaviors are derived from the integration of the cognitive, affective, and psychomotor domains. Knowledge, attitudes, and practices could be representatives of the cognitive, affective, and psychomotor domains, respectively. Knowledge refers to the factual, conceptual, procedural, and met cognitive thought [22]. Attitude is an internal or covert feeling and emotion; or selective nature of intended behavior which represents the affective domain. Practice represents the psychomotor domain which refers to the physical movement, coordination and use of motor or neuromuscular activities [22]. Accordingly, participants’ overall knowledge and practice are considered as good if the score is 80% and above; moderate if the score is between 60 and 79%; and poor if the score is less than 60%. Similarly, attitude towards nosocomial infection was assessed using 8 questions. Responses to questions related to attitude were graded on a 3-point Likert scale with an agreement scale ranging from ‘1’ for disagree to ‘3’ for agree [7]. The overall level of attitude was categorized using Bloom’s cut-off point: positive if the score was 80% and above; neutral if the score was 60–79%; and negative if the score was less than 60%.

### Data collection methods

A simple random sampling method and lottery technique were used to select the respondents and a quantitative method of data collection was employed through a self-administered questionnaire. The quantitative method involves assessment of the impact of medical education on the knowledge, attitude, and practices of 422 graduate students on the prevention and control of nosocomial infection.

### Data quality

The data collection instrument format was developed in English by different individuals for its accuracy and desired results. The data collectors used a self-administered questionnaire for graduate students of the Health Sciences, class of 2018.

### Data analysis and interpretation

After receiving a complete response of the questionnaires, data were analyzed using descriptive statistics by IBM SPSS Version 20.0. Demographic characteristics are presented in tabular form using descriptive statistics and reported as mean, median, standard deviation, frequency, and percentage as presented in tables.

### Ethical considerations

The study was conducted after a written ethical clearance is obtained from the ethical research committee of the School of Biomedical Laboratory Sciences and College of Medicine and Health Sciences. Moreover, the consent forms of the participants were completed voluntarily by the study participants themselves.

### Pretest

To evaluate the understandability and applicability of the instruments used, pretest data were collected and checked from 10 medical laboratory graduate students using a self-administered questionnaire.

## Results

Self-administered questionnaires were collected from 422 respondents. Out of these respondents, 32% were female while only 2% of them were above 25 years of age. The proportions of respondents were: (6.9%) health informatics, (6.9%) medical laboratory sciences, (15.6%) health officers, (4.5%) physiotherapy, (4.3%) environmental and occupational health and safety, (6.6%) psychiatry, (5.5%) optometry, (14.7%) midwifery, (17.5%) nursing, (12.3%) pharmacy, and (5.2%) anesthesia graduated students. Only 40% of the respondents had been trained in infection, prevention out of which 39% took the training at least a year ago (Table 4).

**Table 4:** Baseline characteristics of graduate health science students towards prevention and control of nosocomial infection

Characteristics		Frequency	Percentage (%)
Gender			
Male		287	68%
Female		135	32%
Age			
21–25		414	98%
25–30		8	2%
Department			
Health informatics		29	6.9%
Medical laboratory sciences		29	6.9%
Health officer		66	15.6%
Physiotherapy		19	4.5%
Environmental &occupational health & safety		18	4.3%
Psychiatry		28	6.6%
Optometry		23	5.5%
Midwives		62	14.7%
Nurse		74	17.5%
Pharmacy		52	12.3%
Anesthesia		22	5.2%
Start Clinical attachment	2nd	231	54.8%
	3rd	92	21.8%
	4th	52	12.3%
	No attachment	47	11.1%
Attachment last	Month	375	88.9%
	No clinical attachment	47	11.1%
Clinical attachment shift	4 h	62	16.5%
	6 h	95	25.3%
	8 h	102	27.2%
	12 h	64	17.1%
	No shift in case of on clinical attachment	52	13.9%
Training on “infection prevention”	Yes	169	40.0%
	No	253	60.0%
Duration of training	Less than 6 months ago	29	17.2%
	6 months to 1 year	74	43.8%
	More than one year	66	39.0%

### Knowledge of nosocomial infections

**Fig. 2:**
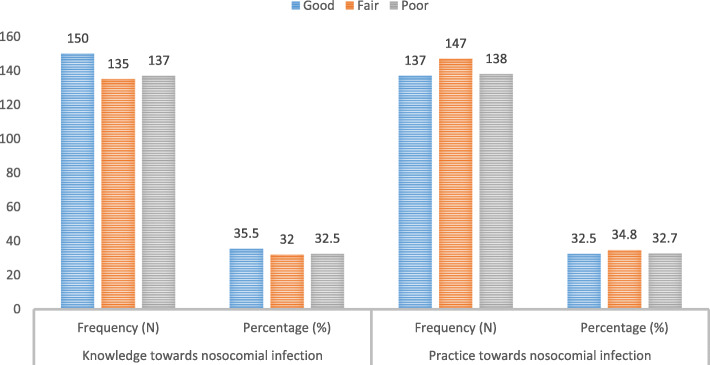
Overall knowledge and practice scores of graduate health science students towards nosocomial infection

Even though 40% of the respondents stated that they had taken training on nosocomial infections, only 35.5% had good knowledge of nosocomial infections (Fig. 2).

From the 55 questions administered, the score of knowledge of the respondents ranged from 11 to 55, with a mean score of 40.64 at std. of + 12.73 (Table 5).

**Table 5:** Assessment of knowledge in graduate health science students towards nosocomial infection

Knowledge regarding nosocomial infection	Poor N (%)	Fair N (%)	Good N (%)
1. I am fully aware of hand-washing guidelines	134 (31.7%)	46 (11%)	242 (57.3%)
2. I have been watching or supervised during hand-washing activity	157 (37.1%)	68 (16.1%)	197 (46.8%)
3. Healthcare facilities harbor a variety of microorganisms that could be transmitted by healthcare workers.	91 (21.5%)	80 (19.0%)	251 (59.5%)
4. I know how to use the Biohazard bag container.	104 (24.6%)	107 (25.4%)	211 (50.0%)
5. I know where and how the contents of Biohazard bags or containers are disposed.	100 (23.7%)	123 (29.1%)	199 (47.2%)
6. I know the safety precautions for disposal of needles, syringes, catheters, etc. and risk of transmission of nosocomial infections to healthcare worker.	84 (19.9%)	76 (18.0%)	262 (62.1%)
7. Nosocomial infections may be transmitted via medical equipment such as syringes, thermometers, needles, catheters, stethoscopes etc.	71 (16.9%)	76 (17.9%)	275 (65.2%)
8. Neutropenic patients or those with communicable diseases (e.g. Diseases of the respiratory system) should be kept in private rooms.	76 (18.0%)	109 (25.8%)	237 (56.2%)
9. Some microorganisms are not eradicated by alcohol-based solutions	58 (13.7%)	96 (22.8%)	268 (63.5%)
10. Hand hygiene should be performed after removing sterile or non-sterile gloves.	90 (21.30%)	54 (12.8%)	278 (65.9%)
11. I am aware of the recommended guidelines for hand hygiene	89 (21.1%)	89 (21.1%)	244 (57.8%)

N indicates number of graduate health science students; % indicates percentage

As reflected in Table 5, out of the total respondents, 59.5% had good knowledge of the modes of transmission and risk factors for nosocomial infections; 57.3% of the respondents also stated that they were fully aware of hand-washing guidelines; 47.2% knew where and how the contents in biohazard bags or containers are being disposed. It is also shown that 65.2% of them knew that nosocomial infections could be transmitted via fomites, and 59.6% of the respondents understood that healthcare facilities harbor a variety of microorganisms that could be transmitted by healthcare workers. Then, 62.1% of respondents were fully aware of safety precautions for the disposal of used medical equipment, and 56.2% of them believed that neutropenic patients like those with diseases of the respiratory system should be kept in private rooms. Furthermore, 57.8% of the graduates were knowledgeable in the use of alcohol-based formulations, and 63.5% of them stated that some microorganisms were not totally removed by alcohol-based solutions.

### Practices for the prevention and control of nosocomial infections

The overall practice scores showed that 32.5% have good practice in the prevention and control of nosocomial infection. The score of the practice of the respondents ranged from 14 to 70, with a mean of 45.61 atstd+ 15.35 (Table 6).

**Table 6:** Assessment of practice in graduate health science students towards the prevention and control of nosocomial infection

Practice regarding nosocomial infection	Poor N (%)	Fair N (%)	Good N (%)
1. I follow recommended guidelines for use of alcohol-based solutions or other antiseptics before and after the work	152 (36.0%)	92 (21.8%)	178 (42.2%)
2. I follow recommended guidelines for use of alcohol-based solutions or other antiseptics before opening vascular access; equipment.	146 (34.6%)	110 (26.1%)	166 (39.3%)
3. Use of alcohol-based solutions or other antiseptics between each patient contact	101 (25.8%)	100 (25.6%)	190 (48.6%)
4. I wash my hands or rub with alcohol-based solution or other antiseptics before and after providing any of work procedure	107 (25.3%)	103 (24.3%)	212 (50.4%)
5. I wash my hands or rub with alcohol-based solution or other antiseptics after contact with equipment objects likely to be contaminated followed by patient care activity e.g. taking vital signs	109 (25.8%)	105 (24.9%)	208 (49.3%)
6. I wash hands before and after drawing and, or manipulating the patient’s body fluid sample	91 (21.6%)	111 (26.3%)	220 (52.1%)
7. I always wash hands before and after having direct contact with a patient’s intact skin	161 (38.2%)	87 (20.6%)	174 (41.2%)
8. I always wash my hands before and after inserting indwelling urinary catheters	134 (31.8%)	77 (18.2%)	211 (50.0%)
9. I always wash my hands when moving from a contaminated body site to a clean-body site during patient care	130 (30.8%)	95 (22.5%)	197 (46.7%)
10. I occasionally polish my fingernails or wear artificial nails	151 (35.8%)	134 (31.7%)	137 (32.5%)
11. I am less compliant with recommended guidelines for reducing transmission of NI when workload increases or in emergencies.	125 (29.6%)	151 (35.8%)	146 (34.6%)
12. I wash my hands after touching inanimate surfaces and objects in the patient’s surroundings	118 28.0%)	111 (26.3%)	193 (45.7%)
13. During a busy patient care episode, I chart or use the computer keyboard with my gloves on	154 (36.4%)	134 (31.8%)	134 (31.8%)
14. I remove my ring, watch or bracelet before beginning hand hygiene.	112 (26.6%)	128 (30.3%)	182 (43.1%)

^a^N indicates the number of graduate health science students; % indicates percentage

Respondents reflection to correctly following guidelines for the use of alcohol-based solutions before and after patient care activities were 42.2%; before opening vascular access equipment were 39.3%; between each patient contact were 48.6%; before and after direct contact with patients’ intact skin were 41.2%; moving from a contaminated body site to a clean body site were 46.7%; before and after drawing or manipulating patient’s body fluid samples were 52.1%; before inserting indwelling urinary catheters were 50.0%; and after touching inanimate objects and equipment in the patients’ room were 45.7%. Of all, 31.8% of the respondents used their computer keyboards with their glove during busy workload. Finally, 43.1% of the respondents removed their rings, watches, or bracelets during hand hygiene (Table 6).

### Attitudes towards the prevention and control of nosocomial infections

**Fig. 3:**
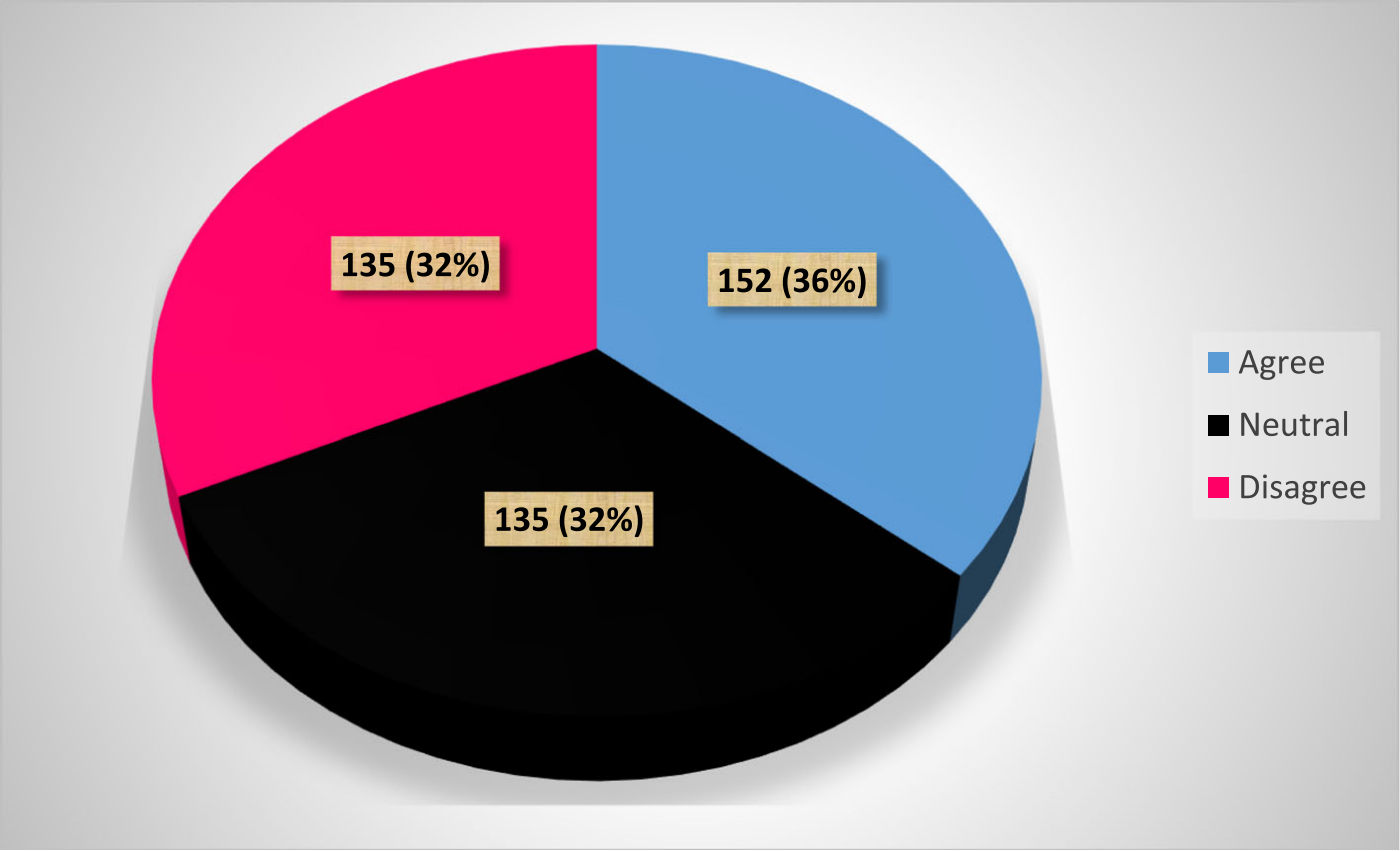
Attitude score of graduate health science students towards the prevention and control of nosocomial infection

The attitudes of students towards the prevention and control of nosocomial infection were 36% (Fig. 3).

Of the total respondents, 61.9% believed that nosocomial infections are posing serious negative outcomes but 44.8% responded the opposite while a colleague is non-compliant with the recommended guidelines for patient safety. Moreover, 51.9% of the respondents regularly used the recommended guidelines and 44.6% believed that health care workers should be sanctioned for noncompliance with the recommended protocols. However, only 48.8% believed to be a role model in demonstrating adherence to the recommended practice for hand hygiene (Table 7).

**Table 7:** Assessment of attitude in graduate health science students towards prevention and control of nosocomial infection

Attitudes regarding nosocomial infection	Disagree N (%)	Neutral N (%)	Agree N (%)
1. In my opinion, nosocomial infections are posing a serious outcome (e.g. Extended hospital stay days, mortality and increased cost of healthcare).	94 (22.2%)	67 (15.9%)	261 (61.9%)
2. In my opinion, I could transmit nosocomial infections.	84 (19.9%)	98 (23.2%)	240 (56.9%)
3. In my opinion, I respond negatively when a colleague (E.g. Nurse or physician) is non-compliant with the recommended guidelines for patient safety.	112 (26.5%)	121 (28.7%)	189 (44.8%)
4. I am more compliant with the recommended guidelines for I am reducing the transmission of a nosocomial infection when training anew worker.	89 (21.1%)	114 (27.0%)	219 (51.9%)
5. I serve as a role model in demonstrating adherence to recommended practices for hand hygiene.	82 (21.0%)	118 (30.2%)	191 (48.8%)
6. It is unrealistic to expect healthcare workers to clean their hands after every contact with the patient.	130 (30.8%)	104 (24.6%)	188 (44.6%)
7. In my opinion, healthcare workers should be sanctioned for non-compliance with protocols for reducing transmission of nosocomial infections (for example, yearly assessment, and denied promotion).	89 (21.0%)	145 (34.4%)	188 (44.6%)
8. In my opinion, healthcare workers should be rewarded (for Example, given plaques, certificate) for compliance with protocols aimed at reducing transmission of nosocomial infections.	88 (20.9%)	98 (23.2%)	236 (55.9%)

^a^N indicates the number of graduate health science students; % indicates percentage

## Discussion

Nosocomial infection is one of the most important challenges in health institutions. Therefore, this study assessed the knowledge, attitude, practice, and associated factors of infection prevention among health science graduate students. The overall score of knowledge (35.5%) was lower than the study conducted in the USA (93%) and Nepal (97%) [16, 23]. Similarly, 59.6% had good knowledge of their etiology, modes of transmission, and risk factors of nosocomial infections which were also lower than the study conducted in New Jersey, USA (95.7%), and Nepal (82%). Moreover, only 59.6% of the participants knew fomites as transmission factors, which is still lower than the study conducted in the USA (98.9%) [16, 23]. This might be due to a difference in study participants. In the USA, the study participants were registered nurses who were working in health care institutions and they might develop knowledge from their experiences and/or in-service training. However, in this study, the participants were graduate students of which 60% never took training on the prevention and control of nosocomial infections. This shows that not all health science students in this college are taking training before their clinical attachments.

In the findings of this study, only 31.5% had good practice in the prevention and control of nosocomial infections of which only 50.4% followed the guidelines for the use of alcohol-containing hand sanitizer which is lower than the study conducted in the USA (78%), but higher than a study conducted in China (11%) [16, 17]. This may be due to the difference in study participants, the accessibility of alcohol-containing hand sanitizer, and the large difference in course curriculum where infection prevention might not be incorporated in all of the target population. Possibly for similar reasons, the attitude of study participants towards the prevention and control of nosocomial infection (36%) was lower than a study carried out in the USA (79.66%) and Nepal (66.0%) [16, 23].

A 52.2% positive attitude towards following the recommended guidelines for reducing the transmission of nosocomial infections was lower than a study conducted among health care workers at Addis Ababa in Ethiopia (83.3%) [18]. This might be due to a difference in study participants in Addis Ababa, where they were registered health care workers who were working in the health institution and they might develop knowledge, attitude, and practice either through their experience or in-service training. However, in this study, the participants were graduate students who reported that 60% of them never took training on the prevention and control of nosocomial infections before their clinical attachment.

Generally, more than half of the respondents had poor knowledge, attitude, and practice on nosocomial infection and the application of infection and prevention procedures.

## Conclusion

The medical education system is the most important and effective tool to bring a better outcome for controlling and preventing nosocomial infections. Incorporating the necessary knowledge into the regular course curricula, organizing training modules to medical students before starting clinical attachment, providing different guidelines and standard operating procedures are also helpful in understanding the nature of infections and how, when, and where to prevent and control nosocomial infection. Therefore, this study showed that a smaller number of respondents had taken infection prevention training on their regular medical system. Consequently, smaller proportions of them had good knowledge, attitude, and practice on the nature of the infection, prevention, and control strategies for nosocomial infections. Therefore, to improve the level of knowledge, attitude, and practice of students towards nosocomial infections, strengthening the medical education system through relevant seminars including short and long-term training is essential. At the same time, the availability of infection prevention guidelines, standard operating procedures, and personal protective equipment like alcohol-based solutions in health institutions are important.

## Recommendations

- Departments, Schools, and College administrative officers should work together to facilitate infection prevention training programs for all health science students before starting their clinical attachments.
- The Ministry of Health and Ministry of Education should work to enforce the universities to incorporate infection prevention knowledge into the course curricula for all health science students.
- All health care institutions must be prepared to give vaccination of common hospital-acquired diseases by making available infection prevention materials and standard operational procedures guidelines at each section of the institution.

## Data Availability

All data generated or analyzed during this study are included in this article.

## Abbreviations

HAIs: Hospital-acquired infections
HCWs: Health care workers
HSS: Health science student
PEP: Post- Exposure Prophylaxis
